# Pediatric Syncope: An Examination of Diagnostic Processes, Therapeutic Approaches and the Role of the Tilt Test: Insights from an 18-Year Single-Center Experience

**DOI:** 10.3390/children12040459

**Published:** 2025-04-03

**Authors:** Serra Karaca, Doruk Özbingöl, Pelin Karaca Özer, Mustafa Lütfi Yavuz, Kemal Nişli

**Affiliations:** 1 Pediatric Cardiology Department, Istanbul Faculty of Medicine, Istanbul University, Istanbul 34093, Türkiye; doruk.ozbingol@istanbul.edu.tr (D.Ö.); kemal.nisli@istanbul.edu.tr (K.N.); 2 Cardiology Department, Istanbul Faculty of Medicine, Istanbul University, Istanbul 34093, Türkiye; pelin.ozer@istanbul.edu.tr (P.K.Ö.); mustafayavuz94@istanbul.edu.tr (M.L.Y.)

**Keywords:** syncope, vasovagal, tilt table test, beta-blockers

## Abstract

**Objectives:** Syncope is a common cause of the transient loss of consciousness, with neurally mediated syncope (NMS) and particularly vasovagal syncope (VVS) being the most prevalent types among older children and adolescents. VVS is primarily caused by heightened parasympathetic activity triggered by emotional or postural stimuli, resulting in a temporary disruption of circulation. Although anamnesis and physical examination play key roles in diagnosing VVS, additional diagnostic methods are necessary in unclear cases. This study aims to evaluate the long-term outcomes of pediatric patients with syncope, focusing on clinical characteristics, diagnosis, and treatment approaches. **Methods:** A retrospective analysis was conducted on 455 pediatric patients aged 8–21 years who presented with syncope at our cardiology clinic between 2005 and 2023. Patients diagnosed with cardiac syncope, epilepsy, or postural orthostatic tachycardia syndrome (POTS) were excluded. The remaining 283 patients were categorized into two groups: those with confirmed VVS—based on a comprehensive evaluation, including medical history, physical examination, and electrocardiography—and those suspected of VVS who lack a confident diagnosis after an initial assessment requiring tilt table testing. Clinical features, diagnostic methods, and treatment outcomes were analyzed. **Results:** The study cohort had a mean age of 13.5 ± 1.6 years, with a female predominance of 69%. Among patients who underwent tilt table testing (TTT), 74.8% exhibited a positive response, with mixed-type syncope being the most prevalent (51%). Syncope recurrence was significantly higher in the TTT group (54%) compared to the clinically diagnosed group (15%) (*p* < 0.001). Relapse risk was strongly associated with the syncope subtype, particularly cardioinhibitory type 2B (OR: 2.3, 95% CI: 1.1–4, *p* < 0.01), and episode frequency (OR: 1.7, 95% CI: 1.3–2.5, *p* = 0.03). Beta-blocker therapy was selectively administered and demonstrated a reduced relapse risk in a univariate analysis. **Conclusions:** VVS is a significant health issue in pediatric patients and the therapeutic modalities available encompass various interventions, including modifications to lifestyle, adequate hydration, and pharmacological therapies. TTT was found to be an effective diagnostic tool for identifying high-risk patients and is recommended for appropriate cases in pediatric VVS diagnosis in accordance with the guidelines, with the objective of refining therapeutic methodologies and ultimately augmenting patient prognoses.

## 1. Introduction

Syncope is a leading cause of the transient loss of consciousness in pediatric patients. The primary classifications of syncope include neurally mediated syncope (NMS), cardiac syncope (CS), and unexplained syncope [1]. NMS, which encompasses vasovagal syncope (VVS), represents the most common etiology among older children and adolescents. It poses significant health concerns, negatively impacting quality of life and increasing the risk of injury [2]. The underlying pathophysiological mechanism of VVS is an exaggerated parasympathetic response, which may be triggered by emotional stimuli, postural changes, or situational factors [3,4]. The most prevalent subtype is postural VVS, which should be distinguished from orthostatic syncope caused by orthostatic hypotension [5]. The primary pathogenesis of VVS involves a transient reflex-mediated disruption of circulatory homeostasis [6].

Syncope is a common condition, with approximately 15% to 25% of children experiencing at least one syncopal episode before reaching adulthood [7,8,9]. A thorough patient history and a comprehensive physical examination are critical for diagnosing VVS. However, in cases where a definitive diagnosis cannot be established based on clinical history and physical findings alone, additional diagnostic methods become necessary [10].

Syncope represents a significant pediatric health concern with diverse etiologies, necessitating accurate diagnosis and appropriate management strategies [2]. This study aims to assess the long-term outcomes of pediatric patients presenting with syncope at our clinic, with a particular focus on clinical characteristics, diagnostic processes, and treatment approaches.

## 2. Materials and Methods

**Figure 1:**
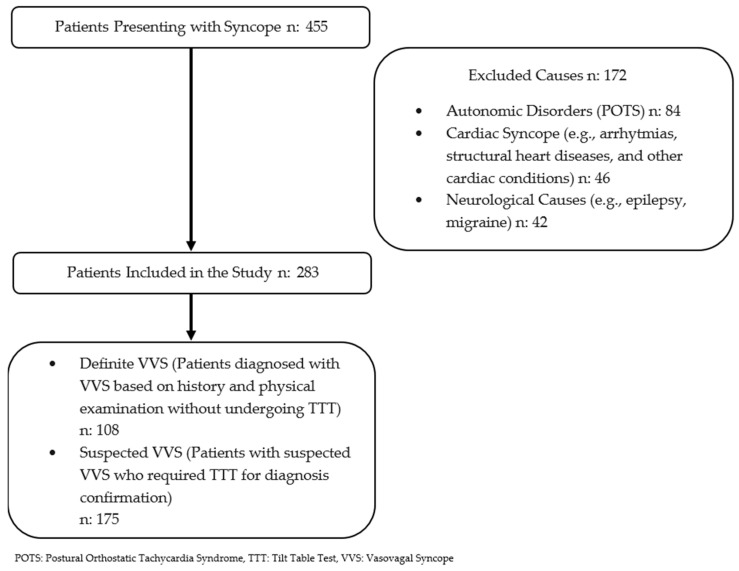
Flow diagram.

This retrospective study evaluated the long-term outcomes of pediatric patients referred to our cardiology department for syncope between January 2005 and February 2023. A total of 455 patients aged 8 to 21 years, with complete follow-up data, were included in the analysis. The overall incidence of syncope was recorded, and cases with a confirmed diagnosis of cardiac syncope, epilepsy, or postural orthostatic tachycardia syndrome (POTS) were excluded from further evaluation. Following these exclusions, the remaining patients were stratified into two distinct groups (Figure 1):

1. Definite VVS group: patients who were definitively diagnosed with vasovagal syncope based on a comprehensive evaluation, including medical history; physical examination; electrocardiography; and, when necessary, echocardiography, brain MRI, and electroencephalography.
2. Suspected VVS group: patients who lack a confident diagnosis of VVS after the initial assessment and required tilt table testing.

A comparative analysis was conducted between these groups to assess differences in diagnostic approaches, treatment strategies, and long-term follow-up outcomes.

### 2.1. Diagnostic Methods

#### 2.1.1. Electrocardiography (ECG)

ECG was performed for all patients presenting with syncope. For patients with a confirmed diagnosis of VVS, ECG assessments were conducted at each follow-up visit to monitor heart rate (HR), atrioventricular (AV) block, and other rhythm disturbances. Additionally, ECG was routinely utilized for monitoring patients receiving pharmacological treatment.

#### 2.1.2. Twenty-Four-Hour Holter Monitoring

Holter monitoring is a frequently employed diagnostic tool in the differential diagnosis of syncope, particularly when arrhythmia-related syncope is suspected. This method is particularly beneficial for patients presenting with clinical or ECG findings suggestive of arrhythmic syncope.

#### 2.1.3. Tilt Table Test (TTT)

TTT, initially introduced by Kenny et al. in 1986, remains a crucial diagnostic tool for evaluating syncope in appropriate candidates [11]. According to the European Society of Cardiology (ESC) guidelines, TTT is indicated in selected cases to clarify uncertain diagnoses [12,13].

In this study, TTT was performed when a definitive diagnosis of VVS could not be established based on clinical history and initial assessments. Our institution adheres to the Brignole et al. protocol for tilt testing [12]. The test is conducted in a controlled environment with access to resuscitation equipment and medications.

#### 2.1.4. TTT Protocol

Patients were positioned supine on the tilt table for 10 min before the test commenced.

The continuous monitoring of HR and rhythm was performed, while blood pressure was recorded at two-minute intervals. In the presence of symptoms, blood pressure measurements were taken every 30 s.

The table was then tilted to a 70° head-up position for 30 min.

If syncope symptoms developed, the test was immediately terminated, and the patient was returned to the supine position.

For patients who did not develop symptoms within 30 min, 400 mcg of sublingual isosorbide dinitrate (ISDN) was administered, and monitoring continued for an additional 15 min or until the symptoms appeared.

The test was considered positive if syncope or presyncope symptoms occurred along with a sudden systolic blood pressure drop of >40 mmHg and/or bradycardia (HR < 50 bpm).

#### 2.1.5. Classification of TTT Responses

TTT responses are categorized into three primary types based on hemodynamic and HR changes during syncope episodes.

- Type 1: Mixed Response

This is the most common form of vasovagal syncope, characterized by a combination of vasodepressor (blood pressure drop) and cardioinhibitory (HR drop) components. The HR does not fall below 40 bpm for more than 10 s. Notably, asystole may occur but lasts less than three seconds. A distinguishing feature of this response is that the blood pressure decline precedes the HR reduction.

- Type 2: Cardioinhibitory Response

This type is primarily defined by a marked reduction in HR, potentially resulting in asystole. It is further divided into two subtypes:

Type 2A (without asystole) is characterized by an HR drop below 40 bpm for more than 10 s without complete asystole. The decline in blood pressure precedes the HR drop.

Type 2B (with asystole) is defined by asystole lasting longer than three seconds. In this subtype, the blood pressure may decrease either before or simultaneously with the HR decline.

- Type 3: Pure Vasodepressor Response

This subtype is characterized by a significant drop in blood pressure without notable changes in HR. HR remains within 10% of baseline values, even at the peak of syncope. Key features include minimal HR variation and marked hypotension resulting in syncope. This response is primarily attributed to excessive vasodilation or a reduction in vascular resistance.

This classification provides a structured approach to interpreting TTT findings, aiding in the differentiation of syncope subtypes and guiding appropriate management strategies.

- Postural Orthostatic Tachycardia Syndrome (POTS)

POTS was diagnosed based on standardized standing test criteria. The diagnostic threshold included an HR increase of at least 40 bpm within 10 min of assuming an upright posture. Additionally, age-specific HR cutoffs were applied, with a maximal HR of ≥130 bpm in children aged 6 to 12 years and ≥125 bpm in adolescents aged 13 to 18 years. These parameters were utilized to ensure a consistent and objective approach to the identification of POTS within the study population [14].

### 2.2. Statistical Analysis

Statistical analyses were conducted using SPSS software version 26.0 (IBM Corp., Armonk, NY, USA). The normality of data distributions was assessed using the Kolmogorov–Smirnov test. For normally distributed (parametric) data, comparisons between groups were performed using Student’s *t*-test, while non-parametric data were analyzed using the Mann–Whitney U test. Pairwise comparisons were performed using the Mann–Whitney U test after the Kruskal–Wallis test. To adjust for multiple comparisons and reduce the risk of type 1 errors, Bonferroni correction was applied by multiplying the original *p*-values by the number of comparisons. After the Bonferroni adjustment, *p*-values of <0.05 were considered statistically significant. Factors influencing syncope recurrence were evaluated using a logistic regression analysis. A stepwise logistic regression was performed to identify the independent predictors of syncope recurrence. To reduce overfitting and stabilize the odds ratio (OR) values, bootstrapping with 5000 resamples was applied to the final multivariate model. The bias-corrected accelerated confidence intervals (95% BCa CI) are reported.

## 3. Results

**Figure 2:**
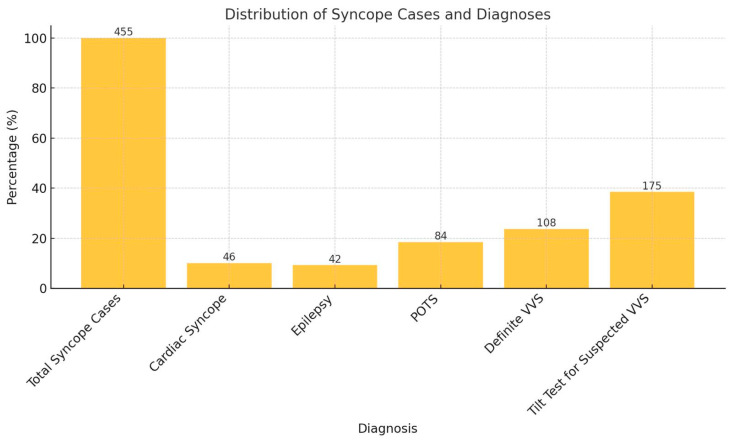
Distribution of syncope cases.

A total of 455 pediatric patients presenting with syncope were evaluated in this study. Of these, 172 patients with cardiac, neurological, and autonomic disorders were excluded from further analysis (Figure 2).

A total of 46 patients (10.1%) were diagnosed with cardiac syncope and excluded from this study. The diagnostic process included a detailed examination of the patient’s medical history, physical condition, and ECG. In cases where structural heart disease was suspected, an echocardiographic evaluation was also performed. The most common cardiac causes included primary arrhythmias, which were observed in 33 out of 46 patients (71.7%) and consisted of supraventricular tachycardia (SVT), ventricular tachycardia (VT), long QT syndrome, Wolff–Parkinson–White syndrome, frequent premature ventricular beat, Mobitz type 2 AV block, advanced type 2 AV block, complete atrioventricular (AV) block, and sick sinus syndrome. We also excluded patients who did not have genetic analyses and who had borderline QTc values. Additionally, structural heart diseases were identified in 13 out of 46 patients (28.3%), including cardiomyopathies (hypertrophic, dilated, non-compaction, and unclassifiable), aortic stenosis (valvular and non-valvular), pulmonary hypertension, coronary anomalies, and cardiac tumors.

A total of 42 patients (9.2%) were excluded due to epilepsy. These patients were referred to pediatric neurology following an initial suspicion of seizure based on their clinical history. Once diagnosed, they were lost to follow-up as they commenced antiepileptic drug therapy.

A total of 84 patients (18.5%) were diagnosed with POTS and were excluded from the study. The diagnosis was confirmed using a standing test, in which patients met the predefined criteria, including an increase in HR of ≥40 bpm within 10 min of standing and the presence of orthostatic intolerance symptoms such as palpitations, dizziness, blurred vision, chest tightness, or syncope.

Following the exclusion of 172 patients, a total of 283 patients were included in the final analysis. The baseline clinical characteristics of the study population, including age, sex distribution, number of syncope episodes, and monitoring duration, are summarized in Table 1.

In total, 108 patients (38.2%) were clinically diagnosed with vasodepressor syncope without requiring TTT, while 175 patients (61.8%) underwent TTT for diagnostic confirmation. The mean age of the study population was 13.5 ± 1.6 years, with a female predominance of 69%. The median number of syncope episodes at the time of first admission was 3 (range: 1–6) for the entire cohort, which was slightly lower in the clinically diagnosed vasovagal syncope subgroup, which comprised 2 episodes (range: 1–5).

The median follow-up period for the entire cohort was 4 years (range: 1–8 years), while patients in the clinically diagnosed subgroup had a slightly shorter follow-up duration of 3 years (range: 1–6 years). All included patients had a minimum follow-up period of one year after their initial vasovagal syncope diagnosis, ensuring sufficient longitudinal data for the analysis.

The distribution of patients undergoing TTT, including clinical characteristics, treatment initiation, and syncope recurrence rates, is detailed in Table 2.

Among the 175 patients who underwent TTT, 131 patients (74.8%) had a positive test result. The baseline tilt test was positive in 57 patients (32.5%), with an average syncope onset time of 13 ± 4 min. In the remaining 74 patients (42.2%), syncope was induced following the administration of ISDN, with an average syncope development time of 6.8 ± 1.8 min. A total of 44 patients (25.1%) exhibited negative results in both the baseline and ISDN-stimulated tilt tests, and no adverse effects related to ISDN administration were reported.

Among the 131 patients with positive TTT results, 68 patients (51%) were classified as having mixed-type syncope, 36 patients (27.4%) had vasodepressor-type syncope, 15 patients (11.4%) had cardioinhibitory type 2A syncope, and 12 patients (9.1%) had cardioinhibitory type 2B syncope.

The treatment approach for VVS was determined based on a clinical evaluation, including medical history, physical examination, and ECG. In patients with a confirmed diagnosis of VVS, no additional tests were performed and an initial management strategy was implemented. This consisted of lifestyle modifications, including increased fluid and salt intake, regular physical activity, and the avoidance of triggering factors. A TTT was conducted in patients with an uncertain diagnosis.

For patients with a negative tilt test, no pharmacological intervention was initiated, and they were monitored while adhering to lifestyle recommendations. In cases where the tilt test yielded positive results, lifestyle modifications were reinforced. Despite recent guidelines advising against the routine use of beta-blockers in adults, this approach was selectively applied in our clinical practice, although at a reduced frequency compared to the period before 2009 [13]. Beta-blockers were administered only when the tilt test results did not indicate a vasodepressor type, with metoprolol being the preferred agent, and when the estimated treatment duration was 24 months.

A total of 108 patients who were clinically diagnosed with VVS and did not undergo tilt testing were followed up without initiating medical therapy. Similarly, 44 patients with negative tilt test results and 37 patients with positive tilt test results were monitored without pharmacological treatment. Beta-blocker therapy was initiated in 94 patients who had undergone tilt testing.

Adverse effects related to beta-blocker therapy were rare. Four patients developed asymptomatic sinus bradycardia, while one patient exhibited bronchial hyperreactivity. No other significant side effects were observed.

The use of Fludrocortisone or Midodrine was infrequent and did not significantly vary between groups. Fludrocortisone therapy was initiated in two patients, while Midodrine was administered in one patient. These three patients had experienced frequent relapses despite beta-blocker therapy and were subsequently diagnosed with cardioinhibitory-type VVS based on tilt test findings.

Syncope recurrence was observed in 110 out of 283 patients (38.8%). Among patients who did not undergo tilt testing, recurrence occurred in 16 patients (15%), whereas 94 patients (54%) in the tilt test group experienced recurrent syncope. Notably, 88% of recurrences occurred within the first year of follow-up.

There were no statistically significant differences in age (*p* = 0.91) or sex distribution (*p* = 0.89) among the groups. However, a significant difference was observed in the initiation of beta-blocker therapy, with 80% of patients in the basal-tilt-positive group and 65% of patients in the ISDN-positive group receiving beta-blockers, whereas no patients in the negative tilt group were started on beta-blockers.

Relapse rates were not significantly different between the groups (*p* = 0.12). However, the distribution of syncope subtypes varied significantly (*p* = 0.017), with mixed syncope being the most common type across all groups. Additionally, vasodepressor syncope was more prevalent in the basal-tilt-positive group (35%) compared to the other groups.

The number of syncope episodes significantly differed between the groups (*p* = 0.008). Patients in the basal-tilt-positive and ISDN-positive groups had a higher mean number of syncope episodes (3.1 ± 0.8 and 3.1 ± 0.9, respectively) compared to those in the negative tilt test group (2.7 ± 0.8). The duration of medication use was similar between the basal-tilt-positive and ISDN-positive groups (21.8 ± 5.1 months vs. 21.4 ± 5 months, *p* = 0.71).

The mean time to syncope occurrence during the basal tilt test was 13 ± 4 min, whereas in the ISDN-induced group, it was 6.8 ± 1.8 min. No significant differences were observed in the overall monitoring period across the groups (*p* = 0.17).

Table 3 summarizes the comparison of patients with and without TTT regarding demographic and clinical characteristics. There were no statistically significant differences in age (*p* = 0.84) or sex distribution (*p* = 0.75) between the two groups. However, patients in the tilt test group had a significantly higher relapse rate (54%) compared to those in the clinically diagnosed group (15%) (*p* < 0.001). Similarly, the initiation of beta-blocker treatment was significantly more frequent in the tilt test group (54%) compared to the clinically diagnosed group (15%) (*p* < 0.001).

The mean number of syncope episodes was significantly higher in the tilt test group (3 ± 0.9) than in the clinically diagnosed group (2.4 ± 0.8) (*p* < 0.001). Additionally, the monitoring period was significantly longer in the tilt test group (4.1 ± 1.2 years) compared to the clinically diagnosed group (2.9 ± 1.1 years) (*p* < 0.001). There was no significant difference in the duration of medication use between the two groups (*p* = 0.25).

A comparative analysis of patients based on the syncope subtype is presented in Table 4A. The results of pairwise comparisons after the Kruskal–Wallis test, including Bonferroni-adjusted *p*-values, are presented in Table 4B.

The baseline clinical characteristics were analyzed across different syncope types, including mixed, cardioinhibitory type 2A, cardioinhibitory type 2B, and vasodepressor syncope. There were no significant differences in age (*p* = 0.4) or sex distribution (*p* = 0.8) across the syncope types.

Beta-blocker use was significantly higher in the mixed, cardioinhibitory type 2A, and cardioinhibitory type 2B groups than in the vasodepressor syncope group (*p* < 0.001 for each). Fludrocortisone and Midodrine use did not show any statistically significant differences between the groups after the Bonferroni corrections (Table 4B).

The relapse rates differed significantly across syncope subtypes. The lowest relapse rate was observed in the mixed syncope group (4%), followed by the cardioinhibitory type 2A group (1%). In contrast, the highest relapse rates were noted in the cardioinhibitory type 2B (60%) and vasodepressor (31%) groups. This difference in relapse rates was statistically significantly higher in the cardioinhibitory type 2B and vasodepressor groups than in the mixed syncope group (*p* < 0.001 for each) (Table 4B).

There were no significant differences in the time elapsed until a positive test result during the baseline tilt test (*p* = 0.5) or after ISDN administration (*p* = 0.6) across the groups. Similarly, the mean monitoring period (*p* = 0.9) and the number of syncope episodes (*p* = 0.2) were comparable among the groups (Table 4A). When the duration of medication use was compared, the cardioinhibitory type 2B group had the longest treatment duration (24.7 ± 4.2 months) and was significantly longer than the mixed group and vasodepressor groups (*p* = 0.032 and *p* = 0.024, respectively) (Table 4B).

The results of the univariate and multivariate regression analyses for syncope relapse are presented in Table 5. Univariate and multivariate regression analyses were performed to identify factors associated with syncope relapse. In the univariate analysis, the initiation of beta-blocker treatment was associated with a reduced likelihood of relapse (OR: 0.3, 95% CI: 0.15–0.71, *p* = 0.004).

The number of syncope episodes demonstrated a strong association with relapse in both univariate (OR: 3.2, 95% CI: 1.4–7.2, *p* = 0.005) and multivariate analyses (OR: 1.7, 95% CI: 1.3–2.5, *p* = 0.03). Similarly, the duration of medication use was a significant predictor of relapse, with consistent findings in both the univariate (OR: 2.2, 95% CI: 1.5–3.2, *p* < 0.001) and multivariate analyses (OR: 2.1, 95% CI: 1.3–3.2, *p* = 0.001).

The type of syncope was also a significant predictor of relapse. In the univariate analysis, vasodepressor syncope (OR: 3, 95% CI: 1.2–7.7, *p* = 0.02) and cardioinhibitory type 2B syncope (OR: 9, 95% CI: 2.5–31.8, *p* < 0.001) were significantly associated with increased relapse risk. In contrast, mixed syncope exhibited a protective effect against relapse (OR: 0.1, 95% CI: 0.03–0.35, *p* < 0.001). The multivariate analysis confirmed a significant association between cardioinhibitory type 2B syncope and relapse, with an OR of 2.3 (95% CI: 1.1–4, *p* = 0.01).

Neither age nor sex was significantly associated with relapse in either the univariate or multivariate analyses (*p* > 0.05).

One patient experienced long-term asystole despite being on beta-blocker therapy, necessitating the implantation of a temporary pacemaker. When the tilt test was repeated, pacing prevented a decrease in heart rate; however, at the sixth minute, a sudden blood pressure drop occurred, resulting in syncope. Fludrocortisone therapy was initiated for this patient, but hypertension developed during follow-up, suggesting an underlying autonomic dysfunction.

## 4. Discussion

In this study, we evaluated children and adolescents who presented to our clinic with syncope between 2005 and 2023. Our analysis included a comprehensive assessment of the differential diagnosis of syncope, the methodologies employed in patient evaluation, the demographic distribution of patients based on etiology, and the long-term follow-up protocols for individuals with both suspected and confirmed VVS.

The initial evaluation of a patient presenting with syncope should include a detailed examination of the patient’s medical history, a comprehensive physical examination, and orthostatic blood pressure measurements, along with a routine ECG. The primary objective of this assessment is to differentiate true syncope from other conditions that cause the transient loss of consciousness [15,16,17]. Conditions such as psychogenic pseudo-syncope, transient ischemic attacks, intoxication, epilepsy, and metabolic disorders must be carefully considered as potential alternative diagnoses.

In cases where the diagnosis is clearly established based on a thorough history examination, a meticulous physical examination, and ECG findings, further diagnostic testing may not be necessary, and therapeutic recommendations can be provided as appropriate [18].

The literature presents a wide range of investigations aimed at comprehensively elucidating and categorizing syncope, given its diverse etiologies, clinical manifestations, and potential consequences. However, the most widely accepted classification system divides syncope into three primary categories: cardiac syncope, reflex syncope, and hypotensive syncope [19].

Among these, VVS is recognized as the most prevalent cause of reflex syncope, particularly in pediatric and adolescent populations [20]. In our study, VVS was identified as the leading etiology of syncope, accounting for 62% of cases, a finding that is consistent with the previous literature [19,20].

The incidence of cardiac syncope varies among studies, with reported rates ranging from 4% to 10% [21]. In our study, cardiac syncope constituted 10% of cases, consistent with previous research conducted in pediatric populations [22]. Among the individuals with cardiac etiologies, arrhythmia was detected in 71.7% of the affected population.

Vasovagal syncope remains a significant clinical concern due to its high prevalence in childhood and its adverse impact on patients’ quality of life. This condition is often triggered by specific factors, such as prolonged standing or exposure to distressing events. The underlying pathophysiological mechanisms of vasovagal syncope are complex and multifactorial. In essence, certain predisposing conditions can provoke aberrant vagal nerve activity, resulting in a rapid decrease in blood pressure (BP); a reduction in HR (bradycardia); excessive sweating (diaphoresis); and a spectrum of symptoms, including blurred vision, dizziness, light-headedness, and nausea, which may ultimately result in syncope [23,24,25,26]. Although comprehensive patient history and thorough physical examinations are emphasized as essential components of the diagnostic process, additional diagnostic methods are often required, with indications guided by established clinical recommendations [2,12,13].

Various TTT protocols are available, including those that do not employ provocative agents, in addition to protocols that utilize isoprenaline, nitroglycerin (GTN), and edrophonium as provocative agents. The use of provocative agents is believed to enhance both the sensitivity and efficacy of the test, as evidenced by high specificity (92–94%) and positive predictive values reported in the literature [27,28,29]. Among these agents, GTN is the most commonly used in clinical practice, demonstrating a sensitivity of 62% and a specificity of 92% [30]. Dindar et al. reported that ISDN-induced tilt tests were effective for the clinical evaluation of patients with syncope, with a sensitivity of 77.5% and a specificity of 91.6% [31].

A study involving 380 adult patients demonstrated that the administration of provocative agents increased the likelihood of a positive TTT result. Moreover, different agents elicited varying test responses; while GTN was predominantly associated with a mixed response, clomipramine more frequently induced a cardioinhibitory response [32]. In alignment with our findings, the tilt test conducted at our institution utilized ISDN as a provocative agent. Consistent with the existing literature, the incidence of positive test outcomes increased following provocation, with the predominant response being mixed.

Various TTT protocols exist, differing in factors such as the tilt angle used during assessment and the duration of the test. The basal or Westminster protocol, which involves positioning patients at a 60° angle for 45 min, has demonstrated a sensitivity of 75% and a specificity of 93% [33]. The duration of the test also varies across different TTT protocols. A study conducted by Stein indicated that extending the test duration beyond 30 min does not enhance the diagnostic accuracy. This study analyzed data from 11 published TTT investigations that utilized the Westminster protocol and compared their findings with a cohort of 213 patients who underwent TTT for 30 to 60 min [34].

Despite the absence of any fatalities reported during TTT, which is recognized as a safe yet occasionally distressing diagnostic procedure, ESC guidelines mandate that resuscitation equipment be readily accessible throughout the TTT procedure [13]. In our study, which spans an extensive 18-year period, no patient fatalities were observed during testing. Furthermore, no critical complications were noted in cases that were excluded from the study due to incomplete documentation.

The therapeutic approach to syncope is primarily determined by its underlying etiology. Various interventions, including lifestyle modifications, physical counter-pressure maneuvers, pharmacological treatments, and pacing techniques, have been employed in the management of reflex syncope. Lifestyle modifications typically involve avoiding situations that may precipitate syncopal episodes, exercise, and ensuring adequate hydration [35].

For many years, beta-blockers were considered the primary therapeutic option. Several small-scale, non-randomized, and uncontrolled studies suggested their efficacy, supporting the hypothesis that beta-blockers mitigate sympathetic activity and prevent exaggerated vagal responses. However, large-scale randomized, long-term, controlled trials evaluating metoprolol, propranolol, nadolol, and atenolol failed to demonstrate significant benefits [36,37,38,39]. Consequently, ESC guidelines do not recommend beta-blockers for the management of reflex syncope [13]. However, all these studies comprise adult studies, and developmental differences in the autonomic nervous system may not be the same in pediatric and adult age groups. Nevertheless, new investigations regarding the efficacy of beta-blockers in the management of vasovagal syncope, as well as the variability in therapeutic responses, have been conducted, and one study has posited that polymorphisms in the beta-1 adrenoceptor result in the activation of receptor signaling and subsequently modify the therapeutic response to beta-blocker therapy [40]. In our study, beta-blocker therapy was associated with a lower likelihood of relapse in the univariate analysis. The most significant predictor of relapse was the number of syncopal episodes at the time of admission. Additionally, prolonged beta-blocker therapy was correlated with an increased relapse rate, further supporting current guideline recommendations. While the correlation between prolonged beta-blocker therapy and increased relapse rates may comprise data supporting the guidelines, the preference for medical treatment in resistant cases with relapse despite lifestyle changes may also explain this result.

Long-term follow-up studies evaluating treatment efficacy in pediatric patients are limited and largely retrospective. In one study involving 97 pediatric patients, Kouakam reported a syncope recurrence rate of 32% [41]. In our study, when considering all patients, regardless of whether they underwent TTT, the overall relapse rate was 38%. However, when analyzed separately, a statistically significant difference emerged: The relapse rate was 15% in the clinically diagnosed group without TTT, whereas it was 54% in the TTT group. The highest relapse rate (60%) was observed in patients with cardioinhibitory type 2B responses. Similarly, Salim et al. found a high relapse rate among patients who underwent TTT [42].

The first study investigating recurrence risk in pediatric VVS patients was conducted by Tao et al. [43]. Their findings suggested that the frequency of prior syncopal events could serve as an indicator of VVS severity. In a retrospective study with an average follow-up of nine months in 42 pediatric patients diagnosed with VVS, they demonstrated that experiencing four or more syncopal episodes before initiating metoprolol treatment was a significant predictor of recurrence [43]. Our study represents the most extensive analysis of TTT outcomes to date, encompassing 175 patients with an 18-year retrospective evaluation and an average follow-up duration of four years. The regression analysis in our study identified the presence of four or more syncopal episodes at admission as a critical threshold for relapse risk, aligning with previous findings.

The use of pacemakers in the management of VVS has been a long-standing subject of debate. In our cohort, no patients required permanent pacemaker implantation for VVS. However, one patient with recurrent syncope despite medical therapy underwent TTT with a temporary pacemaker. Although the pacemaker effectively prevented bradycardia, the patient still experienced syncope due to hypotension.

Clinical criteria that may warrant the consideration of pacing include the absence of prodromal symptoms resulting in falls with injury, frequent recurrent episodes (two to six or more per year), prolonged syncopal episodes associated with abnormal motor activity or urinary incontinence, and the need to continue driving, provided that successful pacing intervention occurred. However, such cases are relatively rare, comprising approximately 1% of patients presenting to specialized syncope clinics annually. Pacing remains a complex therapeutic strategy and has not yet been proven to be a consistently effective treatment for VVS [44].

Since TTT is not a widely used diagnostic method and there is no clear superiority of medical treatment methods, including beta-blockers, it seems important for clinicians to educate patients about lifestyle changes, exercise, and adequate fluid intake in the management of VVS [35].

One of the strengths of this study is the extensive 18-year retrospective analysis, which provides a comprehensive evaluation of long-term trends in syncope management. However, due to the evolving nature of clinical guidelines over this period, variations in patient management approaches were observed. Additionally, while a substantial number of patients were initially considered, some cases had to be excluded due to incomplete data. Despite these limitations, this study offers valuable insights into the long-term outcomes of syncope and contributes to the growing body of literature on this subject.

## 5. Conclusions

Syncope, particularly VVS, stands as a major health concern among children and adolescents, frequently disrupting daily activities and quality of life. Accurate diagnosis and the identification of high-risk patients are paramount to delivering the most effective management. Our findings underscore TTT as a diagnostic modality that is capable of precisely distinguishing syncope subtypes and flagging at-risk individuals. Given its demonstrated safety profile and diagnostic robustness, we advocate for the integration of TTT into pediatric VVS evaluations in appropriate cases, aiming to enhance therapeutic strategies and ultimately improve patient outcomes.

## Figures and Tables

**Table 1:** Baseline clinical characteristics of the study groups.

Variables (n)	Total (283)	Clinically Diagnosed with Vasodepressor Syncope (108)	Undergoing TTT (175)
Age (years)	13.5 ± 1.6	13.5 ± 1.5	13.5 ± 1.6
Sex			
Female	196 (69%)	76 (70%)	120 (69%)
Male	87 (31%)	32 (30%)	55 (31%)
Number of syncope episodes	3 (1–6)	2 (1–5)	3 (1–6)
Monitoring period (years)	4 (1–8)	3 (1–6)	4 (1–8)

**Table 2:** Distribution of patients undergoing TTT.

Variables (n)	Negative TTT (44)	Basal TTT Positive (57)	Positive After ISDN (74)	*p*-Value
Age (years)	13.3 ± 1.9 (8–18)	13.6 ± 1.5 (9–18)	13.6 ± 1.6 (9–17)	0.91
Male sex	15 (34%)	17 (30%)	23 (31%)	0.89
Initiation of beta-blockers	0 ^ab^	46 (80%) ^ac^	48 (65%) ^bc^	<0.001
Initiation of Fludrocortisone	0	0	2, (3%)	0.25
Initiation of Midodrine	0	1 (2%)	0	0.35
Relapse	14 (32%)	11 (19%)	12 (16%)	0.12
Syncope type				0.017
Mixed		29 (51%)	39 (53%)	
Cardioinhibitory type 2A		11 (19%)	4 (5.4%)	
Cardioinhibitory type 2B		7 (12%)	5 (6.6%)	
Vasodepressor		10 (18%)	26 (35%)	
Monitoring period (years)	4 ± 1.3	4.1 ± 1.2	4.1 ± 1.1	0.17
Number of syncope episodes	2.7 ± 0.8 ^ab^	3.1 ± 0.8 ^a^	3.1 ± 0.9 ^b^	0.008
Duration of medication use (months)		21.8 ± 5.1	21.4 ± 5	0.71
Time to positive basal tilt (minutes)		13 ± 4		
Time to positivity after ISDN (minutes)			6.8 ± 1.8	

^a^ Statistically significant difference between negative tilt and positive basal tilt. ^b^ Statistically significant difference between negative tilt and positive tilt after ISDN. ^c^ Statistically significant difference between positive basal tilt and positive tilt after ISDN.

**Table 3:** Comparison of patients with and without TTT.

Variables (n)	Clinically Diagnosed with Vasodepressor Syncope (108)	Undergoing TTT (175)	*p*-Value
Age	13.5 ± 1.5	13.5 ± 1.6	0.84
Male sex	32 (30%)	55 (31%)	0.75
Relapse	16 (15%)	94 (54%)	<0.001
Initiation of beta-blockers	16 (15%)	94 (54%)	<0.001
Duration of medication use (months)	23.4 ± 2.7	21.6 ± 5	0.25
Number of syncope episodes	2.4 ± 0.8	3 ± 0.9	<0.001
Monitoring period (years)	2.9 ± 1.1	4.1 ± 1.2	<0.001

**Table 4:** (**A**) Comparison of patients according to syncope type. (**B**) Bonferroni-corrected *p*-values for pairwise comparisons after the Kruskal–Wallis test.

(A)						
Syncope Type (n)	Mixed (68)	Cardioinhibitor Type 2A (15)	Cardioinhibitor Type 2B (12)		Vasodepressor (36)	*p*-Value
Age	13.5 ± 1.4	13.2 ± 1.7	13.8 ± 1.6		13.9 ± 1.8	0.4
Male sex	21 (31%)	4 (27%)	5 (42%)		10 (28%)	0.8
Initiation of beta-blockers	67 (99%)	15 (100%)	12 (100%)		0	<0.001
Initiation of Fludrocortisone	0	1 (1%)	1 (1%)		0	0.047
Initiation of Midodrine	0	0	1 (1%)		0	0.02
Relapse	3 (4%)	2 (1%)	7 (60%)		11 (31%)	*<0.001*
Time to positive basal tilt (minutes)	12.6 ± 3.9	12.3 ± 3.6	12.9 ± 3.1		15 ± 5	0.5
Time to positivity after ISDN (minutes)	6.6 ± 7.8	7.8 ± 2.6	7 ± 1		6.9 ± 2.2	0.6
Monitoring period (years)	4.1 ± 1	4.2 ± 1.8	4.3 ± 1.4		4 ± 1.1	0.9
Number of syncope episodes	3 ± 0.7	3.4 ± 1	3.6 ± 1.2		3 ± 0.9	0.2
Duration of medication use (months)	21.2 ± 4.7	23.1 ± 5.1	24.7 ± 4.2 ^b^		16.7 ± 5.4	*0.003*
(**B**)						
**Variables**	**p1**	**p2**	**p3**	**p4**	**p5**	**p6**
Initiation of beta-blockers	1	1	<0.001	-	<0.001	<0.001
Initiation of Fludrocortisone	0.12	0.068	-	1	0.46	0.32
Initiation of Midodrine	-	0.068	-	1	-	0.32
Relapse	0.76	<0.001	<0.001	0.056	0.8	0.34
Duration of medication use (months)	0.44	0.032	0.12	1	0.68	0.024

^b^ Statistically significant difference between negative tilt and positive tilt after ISDN. p1: Bonferroni-adjusted *p*-value for the comparison between the mixed and cardioinhibitory type 2A groups. p2: Bonferroni-adjusted *p*-value for the comparison between the mixed and cardioinhibitory type 2B groups. p3: Bonferroni-adjusted *p*-value for the comparison between the mixed and vasodepressor groups. p4: Bonferroni-adjusted *p*-value for the comparison between the cardioinhibitory type 2A and cardioinhibitory type 2B groups. p5: Bonferroni-adjusted *p*-value for the comparison between the cardioinhibitory type 2A and vasodepressor groups. p6: Bonferroni-adjusted *p*-value for the comparison between the cardioinhibitory type 2B and vasodepressor groups.

**Table 5:** Univariate and multivariate analysis for relapse.

Variables	Univariate Regression			Multivariate Regression		
	OR	95% CI	*p*-Value	OR	95% CI	*p*-Value
Age	0.9	0.74–1.13	0.42			
Sex	1.4	0.68–3	0.35			
Initiation of beta-blockers	0.3	0.15–0.71	0.004			
Number of syncope episodes	3.2	1.4–7.2	0.005	1.7	1.3–2.5	0.03
Duration of medication use	2.2	1.5–3.2	<0.001	2.1	1.3–3.2	0.001
Syncope Type						
Mixed	0.1	0.03–0.35	<0.001			
Cardioinhibitory type 2A	0.7	0.15–3.3	0.65			
Cardioinhibitory type 2B	9	2.5–31.8	<0.001	2.3	1.1–4	0.01
Vasodepressor	3	1.2–7.7	0.02			

## Data Availability

The data presented in this study are available on request from the corresponding author. The data are not publicly available due to ethical reasons.

## Abbreviations

The following abbreviations are used in this manuscript:

NMS	Neurally mediated syncope
CS	Cardiac syncope
VVS	Vasovagal syncope
POTS	Postural orthostatic tachycardia syndrome
ECG	Electrocardiography
HR	Heart rate
AV	Atrioventricular
TTT	Tilt table test
ESC	European Society of Cardiology
ISDN	İsosorbide dinitrate
SVT	Supraventricular tachycardia
VT	Ventricular tachycardia
OR	Odds ratio
CI	Confidence interval
BP	Blood pressure
GTN	Nitroglycerin
